# Promoting Healthy Eating Habits for College Students Through Creating Dietary Diaries via a Smartphone App and Social Media Interaction: Online Survey Study

**DOI:** 10.2196/17613

**Published:** 2020-03-31

**Authors:** Masako Watanabe-Ito, Emiko Kishi, Yoko Shimizu

**Affiliations:** 1 Department of Public Health Nursing School of Nursing Toho University Ota, Tokyo Japan; 2 Department of Community Nursing School of Nursing Tokyo Women's Medical University Shinjuku, Tokyo Japan

**Keywords:** health promotion, college students, eating habits, social media, smartphone app

## Abstract

**Background:**

Youth in developed countries face the contradictory health problems of obesity and an excessive desire for weight loss. Developing a better health attitude for college students is essential as this period of life establishes future lifestyle and habits. Online interaction on social media can help to improve eating habits by creating dietary diaries through a smartphone app; however, the effects of such interactions for college students have not been examined to date.

**Objective:**

The aim of this study was to evaluate the potential effectiveness of social media interactions with the use of dietary diaries on a smartphone app to motivate college students in raising self-awareness of their eating habits.

**Methods:**

Forty-two college students in the greater Tokyo area of Japan participated in the study by creating dietary diaries online through a smartphone app and then followed/interacted with each other using social media for 7 consecutive days in September to November 2017. Online surveys were administered at baseline, immediately after creating the dietary diaries, and at 1-month follow up. Participants rated their degree of interest and self-evaluation of eating habits using 7-point scales, and answered multiple choice questions related to their thoughts in choosing meals/drinks among 10 topics. Free descriptions about their overall experience throughout the project were also collected in the follow-up survey.

**Results:**

Data from 38 participants who completed all processes were analyzed. Over time, the mean score for degree of interest in eating habits increased from 4.6 to 6.2 (*P*<.001), while the self-evaluation score decreased from 4.5 to 3.6 (*P*<.001); these significant differences remained after 1 month (5.3, *P*=.002; 4.1, *P*=0.04, respectively). A weak negative correlation (*P*=.009) was observed between scores for degree of interest and self-evaluation. Participants with lower scores for degree of interest at baseline tended to increase their interest level by more than 2 points above the average (*P*<.001). Participants gradually thought more about their eating habits from various perspectives when choosing a meal/drink, particularly with respect to maintaining well-balanced diets and introducing diverse ingredients. Participants evaluated their experiences as interesting/fun and reported familiarity with using the smartphone app and social media as the preferred method to keep track of their eating. All participants welcomed communication with fellow participants on social media and motivated each other, in addition to monitoring their eating habits through online dietary diaries. Some participants experienced difficulty, especially when they were busy or faced a lack of internet access.

**Conclusions:**

Through interactions on social media, college students experienced encouragement and developed an interest and critical thinking with respect to their eating habits. This approach, which embraces peer education and peer support with social media, holds promise for the future of youth health promotion. Further examination will be needed to explore how to sustain this level of heightened awareness.

## Introduction

In the last three decades, lifestyle-related health problems among youth in developed countries have become increasingly complicated given a simultaneous rise in the incidence of obesity and diabetes [1-3] with an excessive desire to lose weight by adopting unbalanced diets [4]. Among youth, college students are a particularly harder group to reach owing to their busy lives taken up by newly available activities associated with college life. Previous studies reported that although college students have adequate nutritional knowledge, their eating behavior is not necessarily healthy because they cannot recognize direct links between eating habits and health [5-8]. Therefore, it is essential to promote healthier eating habits among college students because lifestyles are established during this critical period, which have a significant impact on their future health.

Health education for college students requires new approaches that view young people as managers of their own eating habits rather than as recipients of health information. In Japan, 98.7% of people in their 20s use the internet, 88.7% own smartphones, and 78.5% use social media platforms such as Instagram and Twitter to communicate with each other and obtain information [9]. The World Health Organization also supports the potential of mobile health, which uses mobile and wireless technologies to support the achievement of health objectives, especially for motivating young people to acquire healthy behaviors [10]. Over the past 15 years, many researchers have adopted smartphone apps for health care such as for supporting behavioral management required during mental health care [11] and for self-monitoring in managing long-term conditions [12].

Since the 1980s, paper-based approaches such as food frequency questionnaires [13-15] and single or multiple daily recalls [16-18] have been conventionally applied for dietary assessment. Although these are cost-effective methods, some researchers noted that they are time consuming and rely on participants’ memory and literacy, which can lead to higher rates of underreporting [19,20]. Since 1995, the use of innovative technologies has been shown to improve dietary assessment in various research settings. Research related to the use of dietary diaries with computer-based technologies has been conducted in both personal and interactive situations, demonstrating that data can be collected at a time that is convenient for participants [21,22]. Personal digital assistant technologies provide dietary diaries with a portion size measurement aid to enable participants to easily record their food [23,24], and mobile phone/smartphone-based technologies further enrich the data with the addition of digital photos and voice recording, as well as allowing easier registration regardless of location and time [25-27]. Illner et al [28] conducted a systematic review of the innovative technologies available for dietary diaries, demonstrating that dietary diaries that utilize technology have the potential to be more cost- and time-effective, and utilize less laborious means of data correction.

Public health research has expanded in recent years to explore methods that best promote a healthy diet and the adoption of information and communication technology, including a randomized controlled trial on the typical health specialist-patient relationship [29]. Research to promote college students’ eating habits demonstrated that interventions employing information and communication technology can be effective [30-35], and some researchers adopted smartphones and personal digital assistants as assessment tools [36-38]. However, to date, few studies have examined the effects of online peer communication among participants monitoring their eating habits. Watanabe et al [39] investigated how college students interacted with each other through dietary diaries via an internet weblog that was accessed on flip-style phones; this approach was sufficiently familiar to enable participants to discover new challenges in their eating habits [39]. Turner-Mcgrievy and Tate [40] reported the effect of a weight loss program among adults using social media via smartphones. However, the effects of online interaction on social media by creating dietary diaries through a smartphone app to improve college students’ eating habits have not yet been examined.

Our research explores how interactions through social media and creating dietary diaries with a smartphone app motivate college students to raise self-awareness of their eating habits in an effort to develop effective health education approaches for youth. In this study, we investigated (1) how college students change their interest levels and critical viewpoints toward their eating habits; (2) changes in various viewpoints with regard to their decision-making process when eating; and (3) their experiences from interactions on social media when creating dietary diaries via a smartphone app.

## Methods

### Research Design and Participants

This was a before-after study design conducted from September to November 2017 including 42 college students in the greater Tokyo area of Japan who were recruited through bulletin board posters at 5 cooperating universities. Any students at the cooperating universities under 25 years of age were eligible for inclusion in the study, regardless of gender or living situation. Following completion of all research processes, the participants received 2,000 JPY on prepaid cards that could be used at domestic convenience stores.

### Procedure

#### Overall Design and Grouping

The participants were randomly divided into groups of 3 people and were asked to (1) create dietary diaries through the smartphone app and interact with/follow each other through social media; and (2) answer online surveys at baseline, immediately after creating the dietary diaries, and at 1-month follow up.

#### Creating Dietary Diaries

Participants recorded all of the food and drinks they consumed in their online diaries, including photos, text, and commentary space, during a 7-consecutive day period using a smartphone app. In the diaries, the app allowed them to register what they ate by choosing from a preregistered menu, products, or ingredients. Participants also wrote about their thoughts while eating or drinking. For example, they recorded the type of attention paid to choosing their meal or how they cooked the meal. Participants followed the diaries of their fellow group participants at least once a day, read blog-style diaries, and communicated with each other using the social media function in the commentary spaces of the diaries.

#### Online Surveys

Online surveys were administered three times: at baseline, immediately after the intervention, and at 1-month follow up. Participants were asked to respond to items on: (1) degree of interest in their eating habits, which was rated on a scale of 1 (“not at all”) to 7 (“very much”); (2) self-evaluation of their eating habits on a scale of 1 (“very bad”) to 7 (“very good”); and (3) multiple choice responses to the types of topics they considered when choosing food/drink among 10 topics based on a national survey of health and nutrition in Japan [41]. Data on basic personal characteristics and lifestyle were also collected at baseline. The final follow-up questionnaire included a free-response item asking for their overall experiences through participation in the project.

### Research Settings and Smartphone App

**Figure 1:**
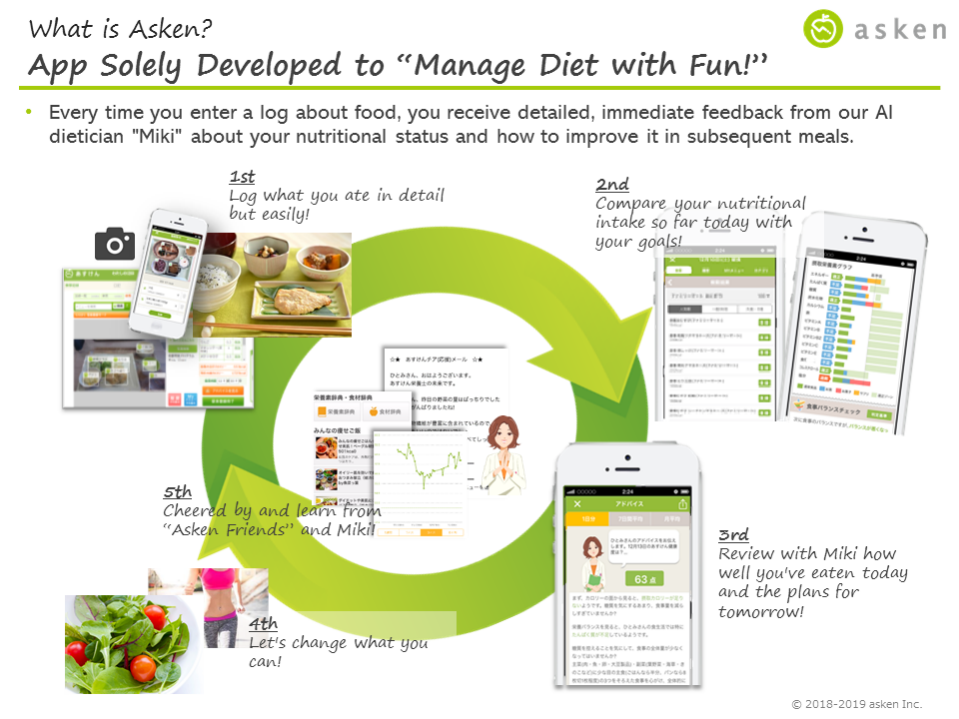
Details of the asken app.

Based on the pretest and our previous research [39], we set a 7-day intervention period so that participants had sufficient time to complete the required tasks, which included both keeping diaries and online surveys, and to ensure that their diaries reflected their eating habits depending on activities on both weekdays and weekends. We determined the number of participants in each group to allow for browsing participants’ diaries without unreasonable effort and to facilitate interaction with each other. We set the number of groups at 14 so that we could feasibly monitor the diaries and preempt any trouble between participants or unexpected disclosures of privacy. The smartphone app asken (asken Inc, Shinjuku, Tokyo, Japan) was used as the medium through which the participants created their dietary diaries and communicated with each other. The asken app is one of the most popular apps for diet management and nutrition improvement, with over 3.5 million users in Japan as of January 2020 [42]. We further selected the asken app for this research because it allows users to create diaries within a social networking system, thereby eliminating the need to send private messages, which safeguarded the participants’ privacy and security (Figure 1).

### Data Analysis

All 42 participants completed the diaries, but 4 were excluded from the analysis because they did not complete the 1-month follow-up online survey.

Quantitative data were analyzed using IBM SPSS Statistics, version 23 (SPSS Inc, Chicago, IL, USA). Changes in points for degree of interest, self-evaluation about their eating habits, and the number of topics that participants considered during their decision making were analyzed at three time points: baseline, immediately after creating dietary diaries, and at the 1-month follow up. First, Friedman tests were applied to determine whether any of the differences between the medians at the three time points were statistically significant. Since the *P* values were less than the significance threshold (*P*<.01), Wilcoxon signed-rank tests were used to examine the results between two time points compared with baseline, and Bonferroni correction was applied to the *P* values for multiple comparisons. To evaluate the degree of coherence between factors of participants who had a greater change in their scores for degree of interest than the average immediately after the intervention, Chi square tests were performed to compare basic characteristics and scores for degree of interest at baseline.

The content of free descriptions was analyzed using the qualitative content analysis method suggested by Graneheim and Lundman [43]. One author (MW) coded the content and discussed the results with other researchers until agreement was reached. The codes were then assigned to suitable categories and subcategories as agreed upon through discussion among researchers.

### Ethical Considerations

This study was approved by the Ethical Review Board at Tokyo Women’s Medical University (approval no. 4055, August 8, 2017). Participants were informed in writing of the research purpose and methods, that participation was voluntary, and that all collected data would be used only for research purposes and would be kept confidential. Participants were instructed not to upload any personal information online and provided written informed consent to participate. The investigators employed the latest security software to prevent data breaches, and all computers and documents were stored in secure areas.

## Results

### Participant Characteristics

The basic characteristics of the participants are summarized in Table 1.

All 38 participants were female, ranging in age from 19 to 22 years. Two participants were nutrition majors, and the remaining 36 majored in other subjects. The majority of participants had a self-reported body mass index in the normal range, whereas about 20% were underweight (mean 20.1, range 17.2-24.1) and none was overweight. The majority of participants lived with their families, followed by living alone and in college dormitories. All participants who lived with their families indicated that their parents cooked their meals, and just over half indicated that they cooked for themselves. The large majority of participants rated themselves to be in “excellent” or “good” health, while 3 participants rated their health as “not so good”; none rated their health as “poor.”

**Table 1:** Basic characteristics of participants (N=38).

Characteristic		n (%)
**Gender**		
	Male	0 (38)
	Female	38 (100)
**Age (years)**		
	19	8 (21)
	20	7 (18)
	21	13 (34)
	22	10 (26)
**Self-reported body mass index**		
	<18.5	8 (21)
	18.5-25	30 (79)
	>25	0 (0)
**Living arrangement**		
	With family	23 (61)
	Alone	11 (29)
	In dormitory	4 (11)
**People who cook daily meals (multiple choice)**		
	Family, especially parents	23 (61)
	Themselves	20 (53)
	Cafeteria	5 (13)
	Eating out	8 (21)
**Self-perceived health condition**		
	Excellent	12 (32)
	Good	23 (61)
	Not so good	3 (8)
	Poor	0 (0)

### Changes in Scores for Degree of Interest and Self-Evaluation of Eating Habits

Participants’ scores for degree of interest in eating habits increased significantly (*P*<.001) while the scores for self-evaluation of eating habits decreased significantly (*P*<.001) throughout the process of creating dietary diaries. These differences remained significant at the 1-month follow up compared with the respective baseline values (Table 2).

The participants’ interest level about their eating increased from baseline immediately after creating their dietary diaries, and then decreased slightly at the 1-month follow up. The average baseline score for self-evaluation of eating habits decreased immediately after creating dietary diaries and increased slightly at the 1-month follow up. A statistically significant but weak negative correlation was observed between scores of interest level and eating habits in self-evaluation (r=0.221, *P*=.009).

Regarding the factors that affected participants who changed their scores for degree of interest by more than the average, a smaller score at baseline (1-2) had a significant effect. However, there were no significant effects according to living arrangement, self-reported body mass index, cooking status, and self-perceived health condition (Table 3).

**Table 2:** Changes of participants’ awareness associated with their eating habits through the intervention (N=38).

Item		Average	Median (range)	*P* value (Friedman test)	*P* value (Wilcoxan signed-rank test)^a^
**Interest level in eating habits^b^**				<.001	
	Baseline	4.6	6 (2-5)		—^c^
	Immediately after the project	6.2	6 (5-7)		<.001
	One-month follow up	5.3	5 (2-7)		.002
**Self-evaluation of eating habits^d^**				.001	
	Baseline	4.5	5 (2-6)		—
	Immediately after the project	3.6	4 (1-7)		<.001
	One-month follow up	4.1	4 (1-6)		.04
**Number of topics considered when choosing a meal/drink^e^**				<.001	
	Baseline	3.7	2 (0-7)		—
	Immediately after the project	6.4	5 (3-9)		<.001
	One-month follow up	5.5	4 (2-8)		.05

^a^Compared with the baseline score after Bonferroni correction.

^b^Scale from 1 (“very bad”) to 7 (“very good”).

^c^Not applicable.

^d^Scale from 1 (“not at all”) to 7 (“very much”).

^e^Multiple choice answers.

**Table 3:** Factors affecting participants with substantial changes in their scores of interest level through the intervention (N=38).

Factor		Difference in points between baseline and after intervention		Chi-square	*P* value^a^
		>2, n (%)	≤2, n (%)		
**Baseline score of interest level**				11.17	.001
	Stronger interest (5-6 at baseline)	2 (5)	14 (37)		
	Weaker interest (1-4 at baseline)	16 (42)	6 (16)		
**Living arrangement**				0.07	.79
	Alone/in dormitories	8 (21)	7 (18)		
	With family	10 (26)	13 (34)		
**Self-monitored body mass index**				0.05	.82
	<18.5	4 (11)	4 (11)		
	≥18.5	14 (37)	16 (42)		
**Responsible for cooking daily meals**				0.01	.94
	Themselves	8 (21)	12 (32)		
	Others (family, cafeteria, eating out)	6 (16)	12 (32		
**Self-perceived health condition**				0.01	.94
	Excellent/good	16 (42)	19 (50)		
	Not so good/poor	2 (5)	1 (3)		

^a^After Yates' correction.

### Changes in Dietary Topics Influencing Decision Making in Eating

The numbers of dietary topics participants thought about when they chose their meal/drink increased immediately after creating dietary diaries compared to that at baseline. The increase and the significant difference was maintained at the 1-month follow up (Table 2).

Considering the details of the topics more carefully (Table 4), at baseline, participants thought about “quantity of food consumed,” “eating a variety of foods/ingredients,” “whether to drink alcohol or not,” and “when to eat.” After creating dietary diaries, the number of participants who chose “the nutrient balance of meals,” “whether to eat breakfast or not,” and “eating a variety of foods/ingredients” increased substantially. Eight participants chose “nothing in particular” at baseline, but no participant chose this response after creating dietary diaries.

**Table 4:** Categories considered during decision making about food and drink based on multiple choice responses (N=38).

Category	Baseline, n (%)	Immediately after the project, n (%)	One-month follow up, n (%)
Nothing in particular	8 (21)	0 (0)	0 (0)
Nutrient balance of meals	4 (11)	31 (82)	25 (66)
Eating a variety of foods/ingredients	14 (37)	23 (61)	24 (63)
Quantity of food consumed	17 (45)	15 (39)	14 (37)
How meals were cooked/processed	5 (13)	6 (16)	7 (18)
Time to eat	8 (21)	15 (39)	14 (37)
Eating breakfast or not	2 (5)	12 (32)	12 (32)
Eating out or not	4 (11)	8 (21)	6 (16)
Eating snacks/junk food or not	7 (18)	13 (34)	9 (24)
Drinking alcohol or not	8 (21)	10 (26)	10 (26)

### Participants’ Experience From the Project

#### Main Categories of Participant Experience

Participants’ descriptions of their project experiences were sorted into 5 categories and 16 subcategories (Textbox 1), along with 52 lower-level codes from 223 meaning units.

#### Category A: “It was Fun/Interesting to Participate in the Project”

All participants responded that it was interesting to participate in the project, and their positive impressions about the project were included in Category A. They indicated that they enjoyed seeing what they and the other participants ate, taking photos, and reading comments and posts from other participants: “It was really fun to see what others ate, because we don’t have a chance to see this in normal life. We are all college students, but live such different lives” (22-year old).

#### Category B: “I Learned From Participating in the Project”

Participants indicated that as a result of keeping their own diaries and observing others’ diaries, they began to pay more attention to their eating habits such as how often they ate snacks, skipped breakfast, and ate/drank late at night: “I hadn’t thought about eating, but through this project, I realized how terrible my eating habits are! How do I eat so many sweets in a day?” (21-year old).

#### Category C: “Participating Caused Some Difficulties”

Some participants wrote that participating was difficult because taking photos, writing comments in their diaries, and following others’ diaries was time consuming and they sometimes forgot to record what they ate. They also wrote that they felt embarrassed to show their diaries to other participants when they did not eat well: “Even though it only took a little time, sometimes it was painful to record my meals in the diary, especially when I needed to do other things, such as study” (20-year old).

#### Category D: “Advantages/Disadvantages of Using the Smartphone App and Social Media”

Many participants mentioned that the smartphone app and social media were an advantage for health education because they were familiar with smartphone apps, and it matched their busy college lifestyles. They also found that using social media enabled them to communicate with each other, allowing them to exchange healthy eating tips such as how to include more vegetables in their meals. They also felt encouraged by other participants through comments and “good” posts to keep going.

Some participants wrote that participation was troublesome when they were without internet access or their smartphone batteries were low. One participant mentioned that it was a pity that they could not upload the project posts to the social media that they normally used to share content with friends. “To look back at our eating habits, using the smartphone app is a good idea! It is very familiar for us because we are living with it. On social media, we learned with each other. They encouraged me to complete it.” (22-year old). “The app needed some time to get used to. Hopefully, it would be better if the recording method becomes easier.” (19-year old).

**Qualitative analysis of participants’ experiences of communication on social media when creating dietary diaries.**

**Category A: “It was fun/interesting to participate in the project”**

A-1: Fun/interesting to keep the records of what I ate.

A-2: Fun/interesting to take photos of what I ate.

A-3: Fun/interesting to see what other members ate.

A-4: Fun/interesting to see comments and stamps from other members.

 

**Category B: “I learned from participating in the project”**

B-1: It made me think more of my eating habits and physical activities.

B-2: It made me more conscious of my eating habits.

B-3: I observed and learned from other members.

 

**Category C: “Participating caused some difficulties”**

C-1: It took time to complete project tasks.

C-2: It was difficult to record in general.

C-3: The period of the project was too short.

 

**Category D: “Advantages/disadvantages of using the smartphone app and social media”**

D-1: Advantages of using the mobile phone app include that it’s familiar, always with me, and easy to record.

D-2: Disadvantages of using the mobile phone app include that it required special techniques and used up batteries.

D-3: Requests to improve the app.

D-4: Would like to use personal social media.

 

**Category E: “My eating habits were affected by This project”**

E-1: Improved eating habits during this project.

E-2: Improved eating habits even after this project.

#### Category E: “My Eating Habits Were Affected by this Project”

Some participants wrote that they changed their eating habits as a result of participating and began to eat breakfast every day, choose well-balanced meals with fresh vegetables/fruits, and avoid too many snacks and midnight eating. “After the project, I am trying to eat well-balanced meals as much as possible with many kinds of ingredients not only carbohydrates such as rice balls and bread” (21-year old).

## Discussion

### Principal Findings

This study represents an early attempt to explain how interaction using social media in conjunction with dietary diaries on a smartphone app motivates college students to develop interest in their eating habits. This intervention involved a multiplex process; nevertheless, the results show that college students experienced encouragement and developed an interest in their eating habits through interaction on social media when creating dietary diaries on the smartphone app. This methodology has potential as an effective means for youth to have a chance to review their eating habits for promoting healthier lifestyles.

### Comparison With Prior Work

#### Participant Characteristics

Participant selection introduced some bias, resulting in only female participants. Young women who were concerned about their eating habits were more likely to be drawn to this research and to agree to participate. This was further influenced by the recruitment method because the collaborating colleges had departments with more female students. A well-developed recruitment plan is needed to enroll similar numbers of male students in future studies. Moreover, participants’ basic characteristics such as self-reported body mass index, living arrangement, who cooked their meals, and their self-perceived health conditions did not differ from the average status of college students in the central-east area around Tokyo [44]. Participants’ self-evaluations indicated that their baseline eating habits were not necessarily ideal, which was consistent with survey results from the Kanto Regional Agricultural Administration Office of Japan Ministry of Agriculture, Forestry and Fisheries [45], which showed that 42.2% of college students who live away from their families do not eat enough vegetables and 71.3% want to improve their eating habits, suggesting the need for a new approach to providing health education on nutrition to this age group.

#### Verifying the Effectiveness of Dietary Diaries and Communication via Social Media: Increased Consciousness of Eating Habits

Participants’ awareness of their eating habits increased, which was maintained during the project, although decreased slightly at the 1-month follow up. Participants became more interested in and thought critically about their eating habits, especially about eating nutrient-balanced meals and a variety of foods/ingredients. A previous study that incorporated use of an iPad reported that using photos and texts to record eating was an effective way to increase awareness of food intake [46]. Similarly, in our study, photos on diaries enabled participants to see at a glance how much they ate and how well-balanced the meal was, complementing their limited written descriptions. In addition, describing their thoughts when choosing a meal/drink in their diaries helped them to recognize what factors affect their decision making. Browsing and writing comments on other participants’ diaries further provided an objective/new point of view to look back their own eating habits in comparative ways. In social learning theory, Bandura and Schunk [47] stated that people learn from each other through observation, imitation, and modeling. Our results support this theory of enhancing the peer-learning process but also suggest two challenges that warrant further examination: (1) how to sustain eating habit awareness/interest for a long period, and (2) how to motivate people to pay attention to the less visible factors such as how meals are processed.

#### Advantage of Using Social Media and Smartphone Apps to Promote Healthy Eating for College Students

The results showed that social media and smartphone apps have great potential for providing health education to youth because these tools are familiar to college students. Most participants evaluated the project as interesting and fun, and indicated that they discovered many tools for improving their eating habits. All participants also indicated that they felt motivated and encouraged by their fellow participants. This experience of peer support is important for health promotion, especially in this age group, as previously reported in the concept analysis of peer health support [48]. Wang and colleagues [49,50] developed a participatory action research strategy called “Photovoice” as an effective method for assessing the needs of social minorities. College students may similarly be seen as a group in need of help, given their lack of interest in and awareness of healthier eating habits. We suggest an approach such as “Photovoice-Online” using social media via smartphones to overcome this disadvantage, which requires participants to meet in person to discuss the problems with each other. Further research is needed to analyze the group dynamics of social media communication depending on specific group characteristics.

When social media is used in research, ethical considerations are an important concern. In particular, privacy and confidentiality safeguards are imperative when adopting social media for use in research. Holmberg et al [51] conducted a study about social media usage of adolescent patients with obesity, stating that social media could be a source for health inspiration, information, and support, but requires competencies. In this study, we adopted social media to encourage college students to be more conscious in their eating habits; however, in the setting of health education, more examination is needed to teach college students health literacy to use social media.

A few participants mentioned that it took time for them to record their meal/drink and communicate with others. Smartphone apps, which are developed and improved at a rapid pace, are already offering photo-based nutrient information, which will make the dietary diary recording process easier and more accurate. As desirable tools for college students, it would be preferable that more complementary smartphone apps including such high-level technologies would be available in the near future.

### Limitations

This study has some limitations. As mentioned earlier, all participants in the study were young women, who are considered to be more conscious of their eating habits than young men. In addition, the study sample had a limited number of participants. Furthermore, as Deliens et al [7] noted, college students’ eating habits are influenced by various factors such as social networks and physical/macro environments. As we were focused on the comparison with national Japanese results, we did not use the established questionnaires for international dietary assessment. Therefore, our results are not generalizable to larger populations. This intervention method adopted a multiplex process; thus, we could not analyze exactly which factors affected participants and to what extent. Finally, we did not examine participants’ group dynamics, which may have also influenced the results.

### Conclusion

This research explores how interactions through social media used in conjunction with smartphone apps of dietary diaries can motivate college students to develop an interest in healthier eating habits. Through interactions on social media when creating dietary diaries on a smartphone app, college students experienced encouragement and developed an interest in their eating. This methodology, which embraces peer education and peer support, holds promise for the future. A closer examination of group dynamics associated with participant interactions and longer-term experiments to develop sustainable motivation are needed to further advance this field of research.
